# Sharp recanalization of extensive multilevel central venous occlusions in a young patient with familial Mediterranean fever: A case report

**DOI:** 10.1016/j.radcr.2026.08.040

**Published:** 2026-09-07

**Authors:** Farah Turkistani, Wiaam Siraj Aldeen, Abdulrahman Hisham Algain, Azzam Khankan

**Affiliations:** a King Saud bin Abdulaziz University for Health Sciences, Jeddah, Saudi Arabia; b National Guard Health Affairs, King Abdulaziz Medical City, Jeddah, Saudi Arabia

**Keywords:** Central venous occlusion, Endovascular recanalization, Sharp recanalization, Stent placement, Venous thromboembolism, Familial Mediterranean fever

## Abstract

Chronic multilevel central venous occlusion (CVO) in young adults is uncommon and presents major challenges in establishing reliable vascular access. Patients with recurrent thrombosis develop extensive collateral formation and progressive loss of venous access. Although familial Mediterranean fever (FMF) is primarily an autoinflammatory disease, emerging evidence suggests an increased risk of thromboembolic events mediated by chronic endothelial inflammation and dysfunction. Endovascular recanalization using conventional and sharp techniques is well described for complex long-segment occlusions; reports specifically describing this approach in a young patient with FMF and recurrent venous thromboembolism, however, remain limited. We report a 23-year-old woman with type 1 diabetes mellitus, hypothyroidism, epilepsy, asthma, and FMF (MEFV E148Q variant), with 3 prior ischemic strokes and approximately 30 venous thromboembolic events despite anticoagulation, in whom an extended inherited thrombophilia workup, including whole-exome sequencing and JAK2 testing, was negative. She presented with chest and abdominal pain, palpitations, dizziness, and limb swelling. Imaging revealed extensive multilevel CVO involving the axillary, subclavian, brachiocephalic, internal jugular, and external iliac veins with prominent collateral formation, and repeated attempts at peripheral and central venous access had failed. Endovascular recanalization was performed under sedation. Conventional wire-and-catheter technique crossed most occlusions; sharp recanalization with a 0.018-inch guidewire was required for the left subclavian vein. Subsequently, sequential balloon angioplasty and stent placement were followed by bilateral placement of 5-French dual-lumen peripherally inserted central catheters (PICCs). Finally, postprocedure venography demonstrated satisfactory recanalization with no major complications. Combined conventional and sharp recanalization restored central venous patency and enabled venous access in extensive multilevel CVO. Both PICC lines remained functional without complication over 7 months of follow up. However, the patient required escalating anticoagulation with an unexplained subtherapeutic anti-Xa level, and CT at 4.5 months showed the subclavian stent in situ without acute pulmonary embolism but persistent chronic left brachiocephalic stenosis with extensive collaterals. This case illustrates both the technical feasibility of endovascular recanalization in this setting and the durability challenge posed by an ongoing, incompletely controlled inflammatory and thrombotic state.

## Introduction

Central venous occlusion (CVO) involving multiple major vessels is most frequently reported in older adults with long-standing indwelling catheters or malignancy [1]. In younger patients, CVO is less common and typically arises secondary to extrinsic compression from malignancy, catheter or device-related thrombosis, or an underlying inherited prothrombotic disorder [1]. Symptomatic CVO often presents with nonspecific findings such as limb or facial swelling, chest discomfort, and dyspnea, resulting from venous hypertension and outflow obstruction [2]. Endovascular therapy (balloon angioplasty with or without stent placement) is considered first-line management for chronic CVO, offering superior short-term efficacy and lower invasiveness compared with surgical reconstruction [[3], [4], [5]]. When conventional wire-and-catheter technique cannot cross a chronic total occlusion, sharp recanalization, which involves forcibly traversing a heavily fibrotic, calcified, or anatomically difficult segment with a needle or stiff wire, is a well-described alternative [6].

Familial Mediterranean fever (FMF) is an autoinflammatory disease not classically considered a hypercoagulable disorder; however, emerging evidence links FMF to an increased risk of venous thromboembolism (VTE), potentially through chronic endothelial activation and procoagulant cytokine release during inflammatory flares [7,8]. Venous thromboembolism and chronic CVO are not themselves rare, and sharp recanalization for chronic CVO is well described in prior series. What has not, to our knowledge, been previously reported is the combination in a young patient of poorly controlled FMF, an extensively negative inherited thrombophilia workup, and a cumulative burden of multilevel central venous occlusion severe enough to exhaust conventional venous access. We describe the endovascular management of this presentation, its 7-month course, and the interplay between the underlying inflammatory disorder and the thrombotic phenotype.

## Case presentation

A 23-year-old woman (81.7 kg) with the history summarized in Table 1 was admitted with an FMF flare despite maximal colchicine and corticosteroid therapy. She developed worsening pressure-like chest pain radiating to the left shoulder that increased with movement, together with abdominal pain, palpitations, dizziness, vomiting, and limb swelling.

**Table 1:** Timeline of relevant clinical history.

Background	About 23-year-old woman (81.7 kg, 159 cm). Type 1 diabetes mellitus, hypothyroidism, epilepsy, asthma, allergic rhinitis, migraine, major depressive disorder, polycystic ovary syndrome, and familial Mediterranean fever (FMF; diagnosed 2022, MEFV E148Q variant) on maximal colchicine and corticosteroids. Three prior ischemic strokes; approximately 30 prior venous thromboembolic events despite anticoagulation. Apixaban failed; warfarin could not achieve target INR at 15 mg; maintained on enoxaparin
Thrombophilia workup	Inherited hypercoagulability workup negative, including whole-exome sequencing and JAK2 testing. PET-CT and normal inflammatory markers argued against systemic vasculitis
About 2 y before presentation	Coronary angiography with venous access via the external jugular vein
About ∼4 mo before presentation	Repeat coronary angiography attempted; femoral access unsuccessful due to difficult vascular anatomy
Preceding hospitalization	Prolonged (approximately 4-mo) admission at an outside institution for candidemia attributed to an infected inferior vena cava (IVC) filter, with left femoral vein thrombosis; IVC filter removed at that institution
Index admission (Oct-Nov 2025)	Admitted with an FMF flare despite maximal colchicine and corticosteroids, with chest and abdominal pain, palpitations, dizziness, vomiting, and limb swelling. Repeated failure of peripheral and central venous access
November 6, 2025	Central venous angioplasty, sharp recanalization, left subclavian stent placement, and bilateral PICC insertion
Follow up to June 2026	Enoxaparin escalated to 120 mg twice daily with persistently subtherapeutic anti-Xa; FMF therapy escalated to anakinra; CT at 4.5 mo showed stent in situ, no acute pulmonary embolism, persistent chronic brachiocephalic stenosis with extensive collaterals

Given recurrent VTE despite anticoagulation, an extended thrombophilia workup was performed. Whole-exome sequencing (WES) and targeted JAK2 testing were both negative, as was the wider inherited hypercoagulability panel; deficiency of adenosine deaminase 2 (DADA2) was specifically considered because of its recognized association with vasculopathy and thrombosis and was not identified on whole-exome sequencing. PET-CT and the absence of elevated inflammatory markers argued against systemic vasculitis. No inherited thrombophilia was therefore identified: her prothrombotic state is best characterized as an acquired phenotype occurring in the context of poorly controlled systemic inflammation, rather than a discrete monogenic thrombophilia. MEFV genotyping demonstrated the E148Q variant.

Anticoagulation had previously been withheld because of recurrent epistaxis, the most recent episode of which was severe and required otolaryngology assessment before resolving. It was resumed as therapeutic enoxaparin 80 mg twice daily (approximately 1 mg/kg twice daily at her weight of 81.7 kg) after new venous thrombi were identified on imaging.

Doppler ultrasound of the lower extremities showed no evidence of DVT on the left, with persistent nonocclusive thrombus in the right common femoral and external iliac veins and associated right leg asymmetry and warmth. Ultrasound of the upper extremities demonstrated non-compressible right subclavian and axillary veins with echogenic intraluminal material, absent color flow, and a monophasic waveform, together with partial thrombosis of the proximal left internal jugular vein; a right brachial PICC line also showed a monophasic waveform on Doppler assessment. Taken together, these findings were consistent with occlusive thrombus extending through the visualized right subclavian and axillary segments, with the monophasic waveforms indicating a more proximal central occlusion not directly visualized by ultrasound; a suspicion subsequently confirmed by CT angiography and catheter venography.

As summarized in Table 1, the patient had undergone repeated central venous access procedures, which together constitute a recognized risk factor for the development of CVO: coronary angiography via external jugular access 2 years earlier, a further attempted coronary angiography approximately 4 months before presentation in which femoral access failed because of difficult vascular anatomy, and multiple prior PICC insertions and exchanges. The patient had also recently completed a prolonged admission at an outside institution for candidemia attributed to an infected IVC filter, complicated by left femoral vein thrombosis; the filter was removed at that institution, and neither the explant culture results nor the primary microbiological records were transferred; we therefore report this diagnosis was made at the referring institution rather than confirmed under our care.

CTA demonstrated severe bilateral brachiocephalic venous stenosis with short-segment chronic occlusions and extensive collateral formation, resulting in profound difficulty obtaining venous access. Multiple attempts at peripheral IV placement without ultrasound guidance failed; a PICC line was ultimately placed under ultrasound guidance after several attempts, but the patient subsequently developed erythema and pain at the insertion site that worsened with movement. She was therefore referred to interventional radiology for central venous angioplasty and PICC line insertion, the primary indication being re-establishment of durable central venous access in a patient in whom conventional access had been exhausted, with treatment of the symptomatic occlusion as a secondary goal.

In preparation for the procedure, anticoagulation was temporarily withheld in view of her history of severe epistaxis, and the associated increased risk of thrombosis was discussed with the patient. We acknowledge that Society of Interventional Radiology consensus guidelines classify venography and venous angioplasty as low-bleeding-risk procedures for which uninterrupted anticoagulation is generally acceptable; the decision here reflected her documented bleeding history rather than the procedural risk itself. Administration of intraprocedural heparin is not recorded in the procedural documentation.

**Fig. 1:**
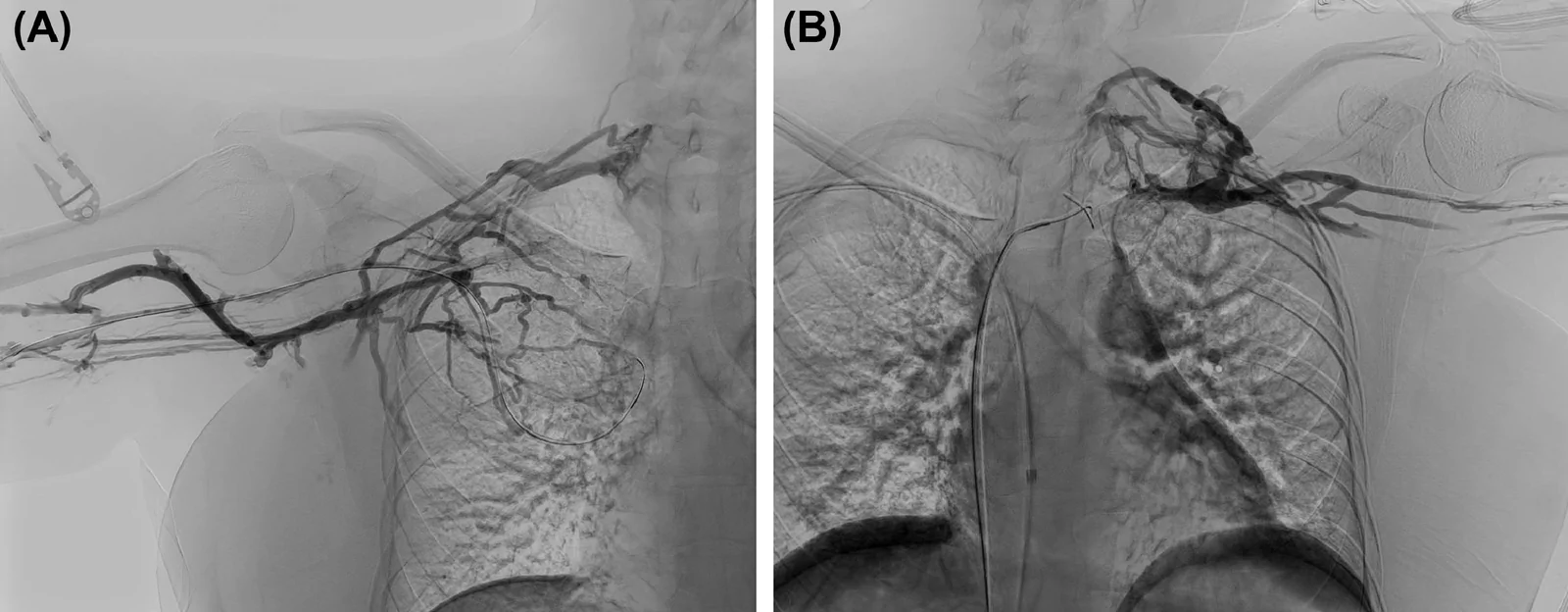
Upper limb venography. (A) Right upper limb: near-complete occlusion of the proximal axillary–subclavian vein with extensive collateralization around the shoulder girdle. (B) Left side: complete occlusion of the proximal subclavian vein with absence of direct antegrade flow beyond the lesion and extensive neck and chest wall collaterals.

The procedure was performed on November 6, 2025 under sedation, with 1% lidocaine for local anesthesia. Venous access was obtained via the bilateral basilic veins, bilateral femoral veins, and right internal jugular vein. Extensive collateral formation was present across all vessels examined. Venography of both arms showed occlusion at the level of the proximal axillary vein with prominent chest wall collaterals draining into the superior vena cava (SVC) (Fig. 1A and B). Internal jugular venography showed occlusion with extensive neck and chest wall collateral channels, and bilateral femoral access confirmed complete occlusion of the external iliac veins with multiple pelvic collaterals.

**Fig. 2:**
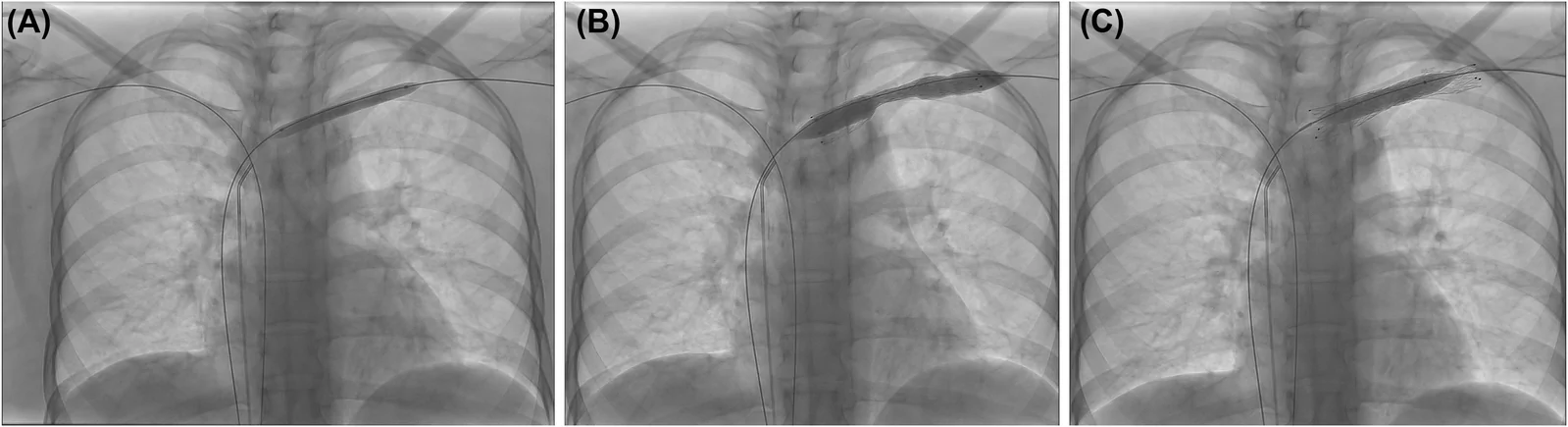
Intraoperative fluoroscopy of the left subclavian vein. (A) Sharp recanalization, with the guidewire traversing the occluded segment and snared into the inferior vena cava via left femoral access. (B) Venovo self-expanding stent deployment. (C) Intra-stent balloon dilatation to achieve optimal vessel expansion.

From the right basilic vein, a 4-French KMP catheter and a 0.035-inch Terumo guidewire (Terumo Corporation, Tokyo, Japan) were advanced under fluoroscopic guidance and crossed the occlusion using a conventional wire-and-catheter technique; moreover, catheter position in the inferior vena cava (IVC) was confirmed by venogram. Balloon angioplasty of the right axillary vein, subclavian vein, and SVC was performed using 6 mm × 60 mm, 7 mm × 60 mm, and 8 mm × 60 mm balloons. Postangioplasty venography showed a small area of contrast extravasation at the axillary vein, controlled with prolonged inflation of an 8 mm × 40 mm balloon at the bleeding site. Using the same technique, the left external iliac vein occlusion was crossed, and angioplasty performed using 7 mm × 60 mm, 8 mm × 60 mm, 8 mm × 40 mm, and 10 mm × 80 mm balloons. The left subclavian vein occlusion could not be crossed with conventional technique and required sharp recanalization; the sharply bent tip of a 0.018-inch guidewire was used to traverse the occlusion and was subsequently snared into the IVC via the left femoral vein, establishing through-and-through access (Fig. 2A). A 0.018-inch guidewire was preferred over a 0.035-inch guidewire, a cut 0.035-inch J-tip, or a bent-needle technique because its finer, stiffer tip permitted controlled and precisely directed penetration of the short, densely fibrotic segment while maintaining coaxial support, and creates a smaller tract should the initial pass prove eccentric. Angioplasty was performed with a 3 mm × 40 mm balloon, followed by placement of a self-expanding Venovo venous stent (BD Interventional, Tempe, Arizona, USA) (Fig. 2B), and intra-stent balloon dilatation with 8 mm × 40 mm and 10 mm × 80 mm balloons (Fig. 2C). After sheath removal from the internal jugular and femoral access sites, hemostasis was achieved with manual compression. The bilateral basilic vein sheaths were exchanged for peelable sheaths, and two 5-French dual-lumen PICC catheters (29.5 cm right and 35 cm left) were advanced centrally with tips positioned at the level of the upper right atrium. Bilateral placement was undertaken to secure durable access in a patient whose peripheral and central options had been exhausted and who required frequent and prolonged intravenous therapy; the specific rationale for 2 dual-lumen catheters rather than 1 is not recorded in the procedure documentation and is addressed further in the Discussion. The catheters were flushed per standard protocol and secured with butterfly fixation devices. The patient tolerated the procedure well, and post-procedure venography confirmed satisfactory recanalization with no immediate complications. Periprocedural antimicrobial prophylaxis is not recorded in the procedural documentation; her candidemia had been treated to completion at the referring institution before transfer, and there was no clinical or laboratory evidence of active infection at the time of stenting.

### Follow-up

The patient was followed for 7 months following the intervention. Anticoagulation was continued with enoxaparin, but achieving therapeutic anticoagulation proved persistently difficult. Shortly before the procedure, she had been maintained on enoxaparin 70 mg twice daily with an anti-Xa level of 0.6 IU/mL. Over subsequent months, the dose was escalated in response to recurrent thrombosis, reaching 120 mg twice daily by January 2026. Despite this, the anti-Xa level was 0.1 IU/mL; unexpectedly subtherapeutic for the dose administered, and discordant with her previously therapeutic levels on lower doses, though she reported full adherence. No cause was identified, and the discrepancy was documented as unexplained. She was admitted for inpatient anti-Xa monitoring, and enoxaparin was continued at the same dose. Anticoagulation was maintained as enoxaparin monotherapy throughout: antiplatelet therapy was considered by the neurology service in view of her prior intracranial infarcts but is not documented as having been initiated during the follow up period, and no antiplatelet agent was prescribed specifically for stent patency.

Chest radiography in January 2026 confirmed the left-sided vascular stent in situ, with mild cardiomegaly and pulmonary congestion. CT pulmonary angiography performed in March 2026, approximately 4.5 months after the intervention, showed interval placement of the left subclavian stent and no evidence of acute pulmonary embolism. It also demonstrated persistent chronic short-segment stenosis of the left brachiocephalic vein with extensive left upper chest, mediastinal, and paravertebral collaterals and opacification of the hemiazygos vein, together with a hypodense filling defect in the distal left brachiocephalic vein suspicious for thrombus. As this study was performed to exclude pulmonary embolism rather than as a dedicated CT venogram, and the left brachiocephalic vein was not optimally opacified, formal assessment of stent and central venous patency was limited.

Both PICC lines remained in situ and functional throughout the follow up period, and no catheter-related complications, infection, symptomatic thrombosis, malposition, or occlusion requiring exchange, were recorded. Dedicated venographic or Doppler surveillance of the stent and catheters was not performed; all follow up cross-sectional imaging during this period was obtained to investigate suspected pulmonary embolism and was negative on each occasion.

In parallel, her FMF remained inadequately controlled. Despite maximal colchicine and corticosteroid therapy, she continued to experience flares, including a severe episode requiring a 1-month admission for fever, abdominal and chest pain, and inflammatory arthritis. Biologic therapy was escalated to anakinra, an interleukin-1 receptor antagonist. At her most recent rheumatology review in June 2026, she was clinically asymptomatic, though her FMF was still classified as uncontrolled.

## Discussion

The most instructive feature of this case is the discordance between an extensive negative inherited thrombophilia workup and a strikingly aggressive thrombotic phenotype. Whole-exome sequencing, JAK2 testing, and the wider hypercoagulability panel were all negative, yet the patient had sustained approximately 30 venous thromboembolic events and 3 ischemic strokes by the age of 23. In the absence of a monogenic explanation, her poorly controlled FMF becomes the most plausible principal driver. Few reports document an association between FMF (primarily an autoinflammatory condition) and susceptibility to thrombus formation, but recent studies show a correlation between FMF and increased VTE risk, plausibly mediated by endothelial activation and procoagulant cytokine release during flares [7,8]. The persistence of thrombotic events alongside ongoing active disease requiring escalation to interleukin-1 blockade is consistent with this proposed mechanism. Her MEFV genotype, the E148Q variant, is of interest in this regard: its pathogenicity has been debated, with some authors regarding it as a benign sequence variant, although recent clinical series support it as a genuine disease-causing variant associated with a generally milder phenotype [9]. The severity of this patient's course therefore illustrates that a variant conventionally associated with mild disease may nonetheless accompany substantial inflammatory and thrombotic morbidity.

The patients repeated central venous access, coronary angiography via the external jugular vein, multiple PICC insertions and exchanges, and an IVC filter that ultimately became infected, represents a second, independent and well-recognized contributor to CVO, and the 2 mechanisms are likely additive. The extent of collateralization seen on CTA is informative; collateral formation reflects the chronicity of venous occlusion, and the eventual need for sharp recanalization at the left subclavian vein is consistent with a long-standing, densely fibrotic occlusion at that site rather than acute thrombus. Technically, conventional wire-and-catheter techniques successfully crossed the right-sided and bilateral external iliac occlusions, whereas sharp recanalization, reserved for occlusions that are heavily fibrotic, calcified, or otherwise resistant to conventional crossing [6], was required only for the left subclavian vein. Although sharp recanalization carries a recognized risk of extravasation, hemothorax, and mediastinal hematoma [10], our patient experienced only a small, self-limited contrast extravasation at the axillary vein, managed with prolonged balloon tamponade.

Reestablishing central venous patency allowed placement of bilateral PICC lines that would not otherwise have been possible. To our knowledge, this is the first report of multilevel endovascular recanalization, including sharp recanalization, in a young patient with poorly controlled FMF, a negative inherited thrombophilia workup, and this degree of multilevel occlusion and collateralization. The 7-month follow up, however, limits any claim of durable success and may be the more clinically relevant lesson. Technical recanalization was achieved, but the underlying disease process was not: the patient required escalation to enoxaparin 120 mg twice daily yet remained subtherapeutic by anti-Xa, follow up CT showed persistent chronic brachiocephalic stenosis with extensive collaterals and a possible new filling defect, and her FMF continued to flare until interleukin-1 blockade was introduced. This trajectory suggests that in inflammation-driven thrombosis, the durability of endovascular recanalization may depend as much on gaining control of the underlying inflammatory disease as on the technical result and supports early consideration of biologic therapy alongside anticoagulation in such patients. It also argues for structured surveillance, using dedicated CT venography or Doppler ultrasound, rather than incidental assessment on studies performed for other indications, in patients at this level of risk.

A further unresolved feature of this case is her apparent failure to achieve therapeutic anticoagulation. Having previously been therapeutic on lower doses, she recorded an anti-Xa level of 0.1 IU/mL while receiving enoxaparin 120 mg twice daily, approximately 1.5 mg/kg twice daily, and reported full adherence. Recognized contributors to apparent low-molecular-weight heparin nonresponse include nonadherence, antithrombin deficiency or consumption, altered volume of distribution, increased renal clearance, and preanalytical factors such as sampling outside the expected peak window; none was established in this patient, and the finding was explicitly documented as unexplained.

This finding is clinically consequential because her recurrent thrombosis occurred despite escalating and apparently adequate dosing, and failure of the anti-Xa level to respond to dose escalation should prompt systematic evaluation rather than continued empiric dose increases. It also reinforces the broader point that, in this patient, thrombotic risk was not adequately controlled by anticoagulation alone.

This report has limitations. Follow up imaging was obtained to investigate suspected pulmonary embolism rather than for dedicated venographic surveillance, therefore stent patency could not be formally assessed. The use of bilateral dual-lumen catheters also warrants consideration, as each indwelling catheter represents an additional potential contributor for thrombosis in a patient at extreme thrombotic risk. In addition, the rationale for placing 2 rather than 1 catheter was not explicitly documented. In retrospect, this decision warranted greater consideration and should not be interpreted as a practice to be adopted routinely. Finally, as a single case, the association between FMF disease activity and thrombotic burden cannot be established definitively.

## Conclusion

**Fig. 3:**
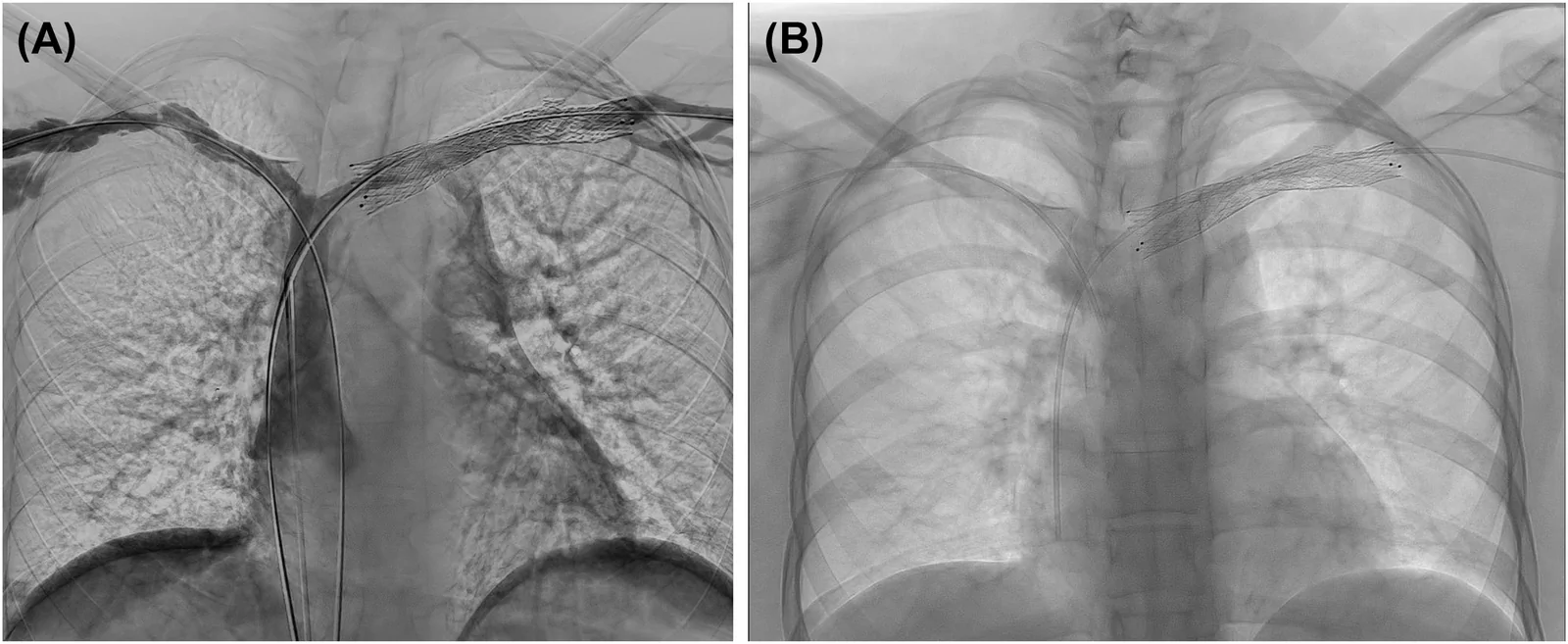
Complete recanalization of the superior vena cava (SVC) and bilateral brachiocephalic veins with balloon angioplasty and stenting (A); bilateral peripherally inserted central catheter (PICC) line insertion (B).

Combined conventional and sharp recanalization successfully restored patency of the bilateral central chest veins and the left femoral and external iliac veins (Fig. 3A), with subsequent placement of bilateral 5-French dual-lumen PICC lines via the basilic veins (Fig. 3B), both catheter tips being sited at the cavoatrial junction/upper right atrium under fluoroscopic guidance. This case demonstrates that extensive multilevel CVO can be recanalized safely and effectively despite exhaustion of conventional venous access. Although, in a patient with active inflammation-driven thrombosis, the technical result alone did not confer durable freedom from recurrence. It underscores FMF as an underrecognized contributor to thrombotic risk and supports pairing endovascular recanalization with aggressive control of the underlying inflammatory disease and structured venous surveillance.

## Ethical approval

Ethical approval was waived by the institutional review board due to the nature of this case report.

## Data availability

The datasets used and/or analyzed during the current study are available from the corresponding author on reasonable request.

## Authors’ contribution

Farah Turkistani contributed to the conception of the study, data collection, and drafting of the manuscript. Wiaam Siraj Aldeen contributed to imaging interpretation and manuscript revision. Abdulrahman Hisham Algain contributed to procedural planning and critical review of the manuscript. Azzam Khankan supervised the study and critically revised the manuscript. All authors read and approved the final manuscript.

## Patient consent

Written informed consent was obtained from the patient for publication of this case report and accompanying images. Consent for publication was obtained for every individual person’s data included in the study. The signed consent form is retained by the authors and is available for review by the Editor upon request.
